# Poly[di-μ_3_-chlorido-di-μ_2_-chlorido-{μ_4_-*N*,*N*,*N*′,*N*′-tetra­kis­[(diphenyl­phosphan­yl)meth­yl]benzene-1,4-diamine-κ^4^ *P*:*P*′:*P*′′:*P*′′′}tetra­copper(II)]

**DOI:** 10.1107/S1600536812010860

**Published:** 2012-03-17

**Authors:** Jia-Qin Liu, Yan Zhang, Ya-Jing Lü, Zhen-Jü Jiang

**Affiliations:** a School of Physics and Chemistry, Xihua University, Chengdu 610039, People’s Republic of China

## Abstract

In the title complex, [Cu_4_Cl_4_(C_58_H_52_N_2_P_4_)]_*n*_, four Cu^II^ atoms are held together *via* two doubly bridging and two triply bridging chlorides, forming a stair-like Cu_4_Cl_4_ core having crystallographically imposed inversion symmetry, while the benzene-1,4-diamine ligand (with a crystallographic inversion center at the centroid) acts in a tetra­dentate coordination mode, bridging two adjacent Cu_4_Cl_4_ cores, resulting in a chain along the *a*-axis direction. One Cu atom has a distorted tetra­hedral geometry, coordinated by one P atom, one μ_2_-Cl and two μ_3_-Cl atoms, while the second Cu atom adopts a trigonal geometry, coordinated by one P atom, one μ_2_-Cl and one μ_3_-Cl atoms.

## Related literature

For the structures and properties of Cu^I^ complexes containing polyphosphine ligands, see: Li *et al.* (2009 ▶); Kohl *et al.* (2006 ▶); Wang *et al.* (2008 ▶); Hou *et al.*(2011 ▶); Ni *et al.* (2011 ▶). For the synthesis of Cu(I) complexes with diphosphine ligands, see: Saravanabharathi *et al.* (2002 ▶); Sivasankar *et al.* (2004 ▶). 
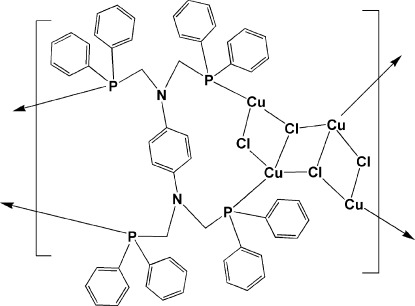


## Experimental

### 

#### Crystal data

[Cu_4_Cl_4_(C_58_H_52_N_2_P_4_)]
*M* *_r_* = 1296.86
Monoclinic, 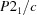

*a* = 10.298 (7) Å
*b* = 17.649 (12) Å
*c* = 18.009 (9) Å
β = 123.94 (3)°
*V* = 2715 (3) Å^3^
*Z* = 2
Mo *K*α radiation
μ = 1.90 mm^−1^
*T* = 296 K
0.20 × 0.15 × 0.13 mm

#### Data collection

Bruker SMART CCD area-detector diffractometer
Absorption correction: multi-scan (*SADABS*; Bruker, 1998 ▶) *T*_min_ = 0.852, *T*_max_ = 1.000
15819 measured reflections
5333 independent reflections
3089 reflections with *I* > 2σ(*I*)
*R*_int_ = 0.085

#### Refinement

*R*[*F* ^2^ > 2σ(*F* ^2^)] = 0.054
*wR*(*F* ^2^) = 0.143
*S* = 0.94
5333 reflections
325 parameters
H-atom parameters constrained
Δρ_max_ = 0.70 e Å^−3^
Δρ_min_ = −0.62 e Å^−3^

### 

Data collection: *SMART* (Bruker, 1998 ▶); cell refinement: *SAINT* (Bruker, 1998 ▶); data reduction: *SAINT*; program(s) used to solve structure: *SHELXS97* (Sheldrick, 2008 ▶); program(s) used to refine structure: *SHELXL97* (Sheldrick, 2008 ▶); molecular graphics: *SHELXTL* (Sheldrick, 2008 ▶); software used to prepare material for publication: *SHELXTL*.

## Supplementary Material

Crystal structure: contains datablock(s) I, global. DOI: 10.1107/S1600536812010860/zq2150sup1.cif

Structure factors: contains datablock(s) I. DOI: 10.1107/S1600536812010860/zq2150Isup2.hkl

Additional supplementary materials:  crystallographic information; 3D view; checkCIF report

## Figures and Tables

**Table 1:** Selected bond lengths (Å)

Cu1—P1	2.1998 (19)
Cu1—Cl2	2.3975 (19)
Cu1—Cl1	2.4140 (17)
Cu1—Cl1^i^	2.565 (2)
Cu2—P2^ii^	2.188 (2)
Cu2—Cl2	2.3062 (18)
Cu2—Cl1	2.3255 (18)

Symmetry codes: (i) 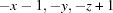; (ii) 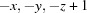.

**Fig. 1.**
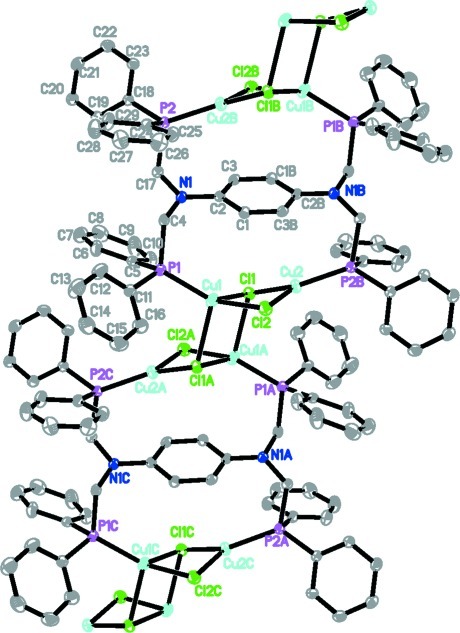
The structure of the title compound with displacement ellipsoids drawn at the 20% probability level. Hydrogen atoms are omitted for clarity. [symmetry code: (A) 1-x, -y, 1-z; (B) -x, -y, 1-z; (C) -1+x, y, z.]

## supplementary crystallographic information

### Comment

Recently, Cu^I^ complexes containing multiphosphine ligands have received much attention so far due to their special structures, novel reactivity, as well as catalytic and luminescent properties (Kohl *et al.*, 2006; Wang *et al.*, 2008; Hou *et al.*, 2011; Ni *et al.*, 2011). However, synthesis of Cu^I^ complexes with tetraphosphine ligands have been virtually not explored, though a great number of Cu(I) complexes with diphosphines were reported (Saravanabharathi *et al.*, 2002; Sivasankar *et al.*, 2004; Li *et al.*, 2009). Herein, we report the synthesis and crystal structure of a new Cu^I^ complex with the tetrakis[(diphenylphosphanyl)methyl]benzene-1,4-diamine ligand (dpppda), the title complex [Cu_4_Cl_4_(dpppda)]_n_.

The asymmetric unit of the title complex, [Cu_2_Cl_2_(C_29_H_26_NP_2_)]_n_, contains two copper ions, two chlorine atoms and one half of the *N*,*N*,*N*',*N*'-tetrakis[(diphenylphosphanyl)methyl]benzene-1,4-diamine ligand (dpppda). Four copper atoms are held together *via* two doubly bridging and two triply-bridging chlorides to form a stair-like Cu_4_Cl_4_ core having a crystallographically imposed centrosymmetry, while the *N*,*N*,*N*',*N*'-tetrakis[(diphenylphosphanyl)methyl]benzene-1,4-diamine ligand (with a crystallographic inversion center at the midpoint of the central phenyl ring) acts as a tetradentate coordination mode to bridge two adjacent Cu_4_Cl_4_ cores resulting in a one-dimentional chain. The structure of the title complex is anologous to the reported complex [Cu_4_I_4_(dpppda)] (Li *et al.*, 2009). Cu1 has a distorted tetrahedral geometry, coordinated by one P atom, one µ_2_-Cl and two µ_3_-Cl atoms, while Cu2 adopts a trigonal geometry, coordinated by one P atom, one µ_2_-Cl and one µ_3_-Cl atoms. The mean Cu—Cl and Cu—P bond distances are 2.40 (1) and 2.19 (2) Å, respectively.

### Experimental

CuCl (0.0198 g, 0.2 mmol) was added with stirring to a solution of *N*,*N*,*N*',*N*'-tetrakis[(diphenylphosphanyl)methyl]benzene-1,4-diamine (0.0900 g, 0.10 mmol) in DMF (5 ml), and the resulting solution was allowed to stir for 1 h at room temperature. Slow diffusion of diethyl ether into the solution give colourless block crystals suitable for X-ray analysis after three days.

### Refinement

All hydrogen atoms were generated geometrically with C—H distances of 0.93Å (aromatic H atoms) and 0.97Å (methylene H atoms) and refined with a riding model with *U*_iso_(H) = 1.2*U*_eq_(C).

### Crystal data

**Table:**

[Cu_4_Cl_4_(C_58_H_52_N_2_P_4_)]	*F*(000) = 1316
*M**_r_* = 1296.86	*D*_x_ = 1.586 Mg m^−^^3^
Monoclinic, *P*2_1_/*c*	Mo *K*α radiation, λ = 0.71073 Å
Hall symbol: -P 2ybc	Cell parameters from 5682 reflections
*a* = 10.298 (7) Å	θ = 2.8–26.3°
*b* = 17.649 (12) Å	µ = 1.90 mm^−^^1^
*c* = 18.009 (9) Å	*T* = 296 K
β = 123.94 (3)°	Block, colourless
*V* = 2715 (3) Å^3^	0.20 × 0.15 × 0.13 mm
*Z* = 2	

### Data collection

**Table:**

Bruker SMART CCD area-detector diffractometer	5333 independent reflections
Radiation source: fine-focus sealed tube	3089 reflections with *I* > 2σ(*I*)
Graphite monochromator	*R*_int_ = 0.085
ω scans	θ_max_ = 26.0°, θ_min_ = 1.8°
Absorption correction: multi-scan (*SADABS*; Bruker, 1998)	*h* = −12→9
*T*_min_ = 0.852, *T*_max_ = 1.000	*k* = −19→21
15819 measured reflections	*l* = −21→22

### Refinement

**Table:**

Refinement on *F*^2^	Primary atom site location: structure-invariant direct methods
Least-squares matrix: full	Secondary atom site location: difference Fourier map
*R*[*F*^2^ > 2σ(*F*^2^)] = 0.054	Hydrogen site location: inferred from neighbouring sites
*wR*(*F*^2^) = 0.143	H-atom parameters constrained
*S* = 0.94	*w* = 1/[σ^2^(*F*_o_^2^) + (0.*P*)^2^] where *P* = (*F*_o_^2^ + 2*F*_c_^2^)/3
5333 reflections	(Δ/σ)_max_ = 0.001
325 parameters	Δρ_max_ = 0.70 e Å^−^^3^
0 restraints	Δρ_min_ = −0.62 e Å^−^^3^

### Special details

**Table:**

Geometry. All e.s.d.'s (except the e.s.d. in the dihedral angle between two l.s. planes) are estimated using the full covariance matrix. The cell e.s.d.'s are taken into account individually in the estimation of e.s.d.'s in distances, angles and torsion angles; correlations between e.s.d.'s in cell parameters are only used when they are defined by crystal symmetry. An approximate (isotropic) treatment of cell e.s.d.'s is used for estimating e.s.d.'s involving l.s. planes.
Refinement. Refinement of *F*^2^ against ALL reflections. The weighted *R*-factor *wR* and goodness of fit *S* are based on *F*^2^, conventional *R*-factors *R* are based on *F*, with *F* set to zero for negative *F*^2^. The threshold expression of *F*^2^ > σ(*F*^2^) is used only for calculating *R*-factors(gt) *etc*. and is not relevant to the choice of reflections for refinement. *R*-factors based on *F*^2^ are statistically about twice as large as those based on *F*, and *R*- factors based on ALL data will be even larger.

### Fractional atomic coordinates and isotropic or equivalent isotropic displacement parameters (Å^2^)

**Table:**

	*x*	*y*	*z*	*U*_iso_*/*U*_eq_	
Cu1	−0.45394 (8)	0.04466 (4)	0.43168 (4)	0.0540 (2)	
Cu2	−0.15464 (7)	0.07255 (4)	0.62081 (4)	0.0487 (2)	
Cl1	−0.30501 (14)	−0.03659 (7)	0.56069 (8)	0.0417 (3)	
Cl2	−0.32846 (15)	0.15805 (7)	0.51454 (9)	0.0526 (4)	
P1	−0.52339 (15)	0.01608 (8)	0.29527 (8)	0.0402 (3)	
P2	−0.07631 (15)	−0.08176 (7)	0.25371 (8)	0.0367 (3)	
N1	−0.2438 (4)	−0.0268 (2)	0.3202 (2)	0.0397 (10)	
C1	−0.1055 (6)	0.0562 (3)	0.4500 (3)	0.0370 (12)	
H1A	−0.1767	0.0948	0.4172	0.044*	
C2	−0.1233 (5)	−0.0133 (3)	0.4094 (3)	0.0336 (11)	
C3	−0.0155 (6)	−0.0698 (3)	0.4619 (3)	0.0364 (11)	
H3A	−0.0251	−0.1175	0.4372	0.044*	
C4	−0.3712 (5)	0.0258 (3)	0.2699 (3)	0.0393 (12)	
H4A	−0.4193	0.0173	0.2064	0.047*	
H4B	−0.3304	0.0771	0.2835	0.047*	
C5	−0.5742 (6)	−0.0830 (3)	0.2617 (3)	0.0430 (13)	
C6	−0.6482 (7)	−0.1100 (4)	0.1736 (4)	0.0658 (18)	
H6A	−0.6962	−0.0752	0.1269	0.079*	
C7	−0.6531 (8)	−0.1842 (4)	0.1535 (5)	0.077 (2)	
H7A	−0.7037	−0.1999	0.0943	0.092*	
C8	−0.5818 (8)	−0.2361 (4)	0.2222 (5)	0.087 (2)	
H8A	−0.5802	−0.2870	0.2094	0.104*	
C9	−0.5123 (8)	−0.2134 (4)	0.3103 (4)	0.088 (2)	
H9A	−0.4684	−0.2490	0.3562	0.105*	
C10	−0.5089 (7)	−0.1380 (3)	0.3292 (4)	0.0647 (17)	
H10A	−0.4619	−0.1230	0.3884	0.078*	
C11	−0.6787 (6)	0.0772 (3)	0.2085 (3)	0.0481 (14)	
C12	−0.7157 (8)	0.0836 (4)	0.1220 (4)	0.078 (2)	
H12A	−0.6607	0.0545	0.1053	0.094*	
C13	−0.8307 (9)	0.1312 (4)	0.0603 (4)	0.099 (3)	
H13A	−0.8583	0.1319	0.0015	0.118*	
C14	−0.9051 (8)	0.1783 (4)	0.0868 (5)	0.086 (2)	
H14A	−0.9836	0.2108	0.0454	0.103*	
C15	−0.8645 (7)	0.1776 (4)	0.1730 (5)	0.0743 (19)	
H15A	−0.9118	0.2110	0.1910	0.089*	
C16	−0.7513 (7)	0.1263 (3)	0.2341 (4)	0.0647 (17)	
H16A	−0.7245	0.1253	0.2927	0.078*	
C17	−0.2353 (5)	−0.0931 (3)	0.2727 (3)	0.0402 (12)	
H17A	−0.3346	−0.0990	0.2156	0.048*	
H17B	−0.2165	−0.1385	0.3077	0.048*	
C18	−0.1096 (5)	−0.1626 (2)	0.1820 (3)	0.0352 (11)	
C19	−0.2520 (6)	−0.1996 (3)	0.1284 (3)	0.0516 (14)	
H19A	−0.3385	−0.1823	0.1271	0.062*	
C20	−0.2680 (7)	−0.2612 (3)	0.0773 (4)	0.0551 (15)	
H20A	−0.3647	−0.2849	0.0418	0.066*	
C21	−0.1420 (7)	−0.2879 (3)	0.0785 (4)	0.0525 (14)	
H21A	−0.1526	−0.3296	0.0441	0.063*	
C22	−0.0008 (7)	−0.2525 (3)	0.1307 (4)	0.0548 (15)	
H22A	0.0849	−0.2699	0.1312	0.066*	
C23	0.0160 (6)	−0.1911 (3)	0.1827 (3)	0.0453 (13)	
H23A	0.1138	−0.1685	0.2190	0.054*	
C24	−0.1553 (6)	0.0012 (3)	0.1797 (3)	0.0430 (13)	
C25	−0.1172 (7)	0.0719 (3)	0.2188 (4)	0.0545 (15)	
H25A	−0.0423	0.0769	0.2798	0.065*	
C26	−0.1918 (8)	0.1364 (3)	0.1661 (5)	0.0701 (19)	
H26A	−0.1672	0.1840	0.1928	0.084*	
C27	−0.2981 (8)	0.1301 (4)	0.0776 (4)	0.0710 (19)	
H27A	−0.3477	0.1732	0.0435	0.085*	
C28	−0.3334 (8)	0.0610 (4)	0.0378 (4)	0.0714 (19)	
H28A	−0.4057	0.0570	−0.0237	0.086*	
C29	−0.2629 (7)	−0.0035 (3)	0.0880 (4)	0.0607 (17)	
H29A	−0.2878	−0.0505	0.0599	0.073*	

### Atomic displacement parameters (Å^2^)

**Table:**

	*U*^11^	*U*^22^	*U*^33^	*U*^12^	*U*^13^	*U*^23^
Cu1	0.0651 (5)	0.0619 (5)	0.0341 (4)	0.0056 (3)	0.0270 (4)	0.0001 (3)
Cu2	0.0389 (4)	0.0554 (4)	0.0406 (4)	−0.0038 (3)	0.0154 (3)	−0.0054 (3)
Cl1	0.0392 (7)	0.0407 (7)	0.0396 (7)	−0.0005 (5)	0.0187 (6)	0.0031 (5)
Cl2	0.0503 (8)	0.0430 (8)	0.0577 (9)	0.0049 (6)	0.0259 (7)	0.0008 (6)
P1	0.0376 (8)	0.0506 (9)	0.0318 (7)	0.0042 (6)	0.0190 (6)	0.0011 (6)
P2	0.0355 (7)	0.0419 (8)	0.0322 (7)	−0.0021 (6)	0.0185 (6)	−0.0041 (6)
N1	0.038 (2)	0.043 (2)	0.035 (2)	0.0018 (19)	0.018 (2)	−0.0083 (18)
C1	0.040 (3)	0.038 (3)	0.032 (3)	0.001 (2)	0.020 (2)	0.004 (2)
C2	0.035 (3)	0.039 (3)	0.029 (2)	−0.002 (2)	0.019 (2)	−0.001 (2)
C3	0.046 (3)	0.031 (3)	0.040 (3)	−0.006 (2)	0.030 (3)	−0.006 (2)
C4	0.041 (3)	0.048 (3)	0.026 (2)	−0.002 (2)	0.017 (2)	0.001 (2)
C5	0.035 (3)	0.055 (3)	0.047 (3)	−0.004 (2)	0.027 (3)	−0.008 (3)
C6	0.066 (4)	0.079 (5)	0.044 (3)	−0.022 (3)	0.026 (3)	−0.010 (3)
C7	0.083 (5)	0.070 (5)	0.087 (5)	−0.034 (4)	0.053 (4)	−0.037 (4)
C8	0.068 (5)	0.058 (4)	0.119 (7)	−0.011 (4)	0.044 (5)	−0.022 (5)
C9	0.088 (5)	0.063 (5)	0.066 (5)	0.000 (4)	0.014 (4)	0.005 (4)
C10	0.064 (4)	0.058 (4)	0.054 (4)	−0.006 (3)	0.021 (3)	0.000 (3)
C11	0.044 (3)	0.064 (4)	0.034 (3)	0.010 (3)	0.021 (3)	0.003 (2)
C12	0.093 (5)	0.090 (5)	0.047 (4)	0.045 (4)	0.037 (4)	0.017 (3)
C13	0.123 (7)	0.107 (6)	0.053 (4)	0.055 (5)	0.041 (5)	0.022 (4)
C14	0.065 (5)	0.106 (6)	0.064 (5)	0.027 (4)	0.021 (4)	0.022 (4)
C15	0.075 (5)	0.072 (5)	0.083 (5)	0.027 (4)	0.048 (4)	0.016 (4)
C16	0.064 (4)	0.070 (4)	0.057 (4)	0.018 (3)	0.032 (3)	0.015 (3)
C17	0.040 (3)	0.047 (3)	0.036 (3)	−0.001 (2)	0.023 (2)	−0.002 (2)
C18	0.038 (3)	0.034 (3)	0.034 (3)	0.000 (2)	0.020 (2)	−0.001 (2)
C19	0.043 (3)	0.058 (4)	0.053 (3)	−0.005 (3)	0.026 (3)	−0.017 (3)
C20	0.052 (4)	0.061 (4)	0.056 (4)	−0.018 (3)	0.033 (3)	−0.022 (3)
C21	0.068 (4)	0.043 (3)	0.056 (4)	−0.006 (3)	0.040 (3)	−0.011 (3)
C22	0.052 (4)	0.053 (4)	0.065 (4)	0.005 (3)	0.036 (3)	−0.007 (3)
C23	0.040 (3)	0.048 (3)	0.050 (3)	0.000 (2)	0.027 (3)	−0.007 (2)
C24	0.043 (3)	0.046 (3)	0.041 (3)	−0.004 (2)	0.025 (3)	−0.004 (2)
C25	0.062 (4)	0.048 (3)	0.053 (3)	−0.011 (3)	0.032 (3)	−0.009 (3)
C26	0.090 (5)	0.035 (3)	0.085 (5)	−0.005 (3)	0.048 (4)	−0.002 (3)
C27	0.077 (5)	0.056 (4)	0.068 (4)	0.006 (3)	0.033 (4)	0.021 (3)
C28	0.077 (5)	0.067 (5)	0.046 (4)	0.000 (3)	0.020 (3)	0.008 (3)
C29	0.074 (4)	0.048 (4)	0.046 (3)	−0.003 (3)	0.025 (3)	−0.004 (3)

### Geometric parameters (Å, º)

**Table:**

Cu1—P1	2.1998 (19)	C10—H10A	0.9300
Cu1—Cl2	2.3975 (19)	C11—C16	1.381 (7)
Cu1—Cl1	2.4140 (17)	C11—C12	1.386 (8)
Cu1—Cl1^i^	2.565 (2)	C12—C13	1.369 (8)
Cu2—P2^ii^	2.188 (2)	C12—H12A	0.9300
Cu2—Cl2	2.3062 (18)	C13—C14	1.382 (9)
Cu2—Cl1	2.3255 (18)	C13—H13A	0.9300
Cl1—Cu1^i^	2.565 (2)	C14—C15	1.364 (9)
P1—C5	1.828 (5)	C14—H14A	0.9300
P1—C11	1.837 (5)	C15—C16	1.399 (7)
P1—C4	1.869 (5)	C15—H15A	0.9300
P2—C18	1.824 (5)	C16—H16A	0.9300
P2—C24	1.836 (5)	C17—H17A	0.9700
P2—C17	1.861 (5)	C17—H17B	0.9700
P2—Cu2^ii^	2.188 (2)	C18—C23	1.381 (7)
N1—C2	1.397 (5)	C18—C19	1.389 (6)
N1—C4	1.442 (6)	C19—C20	1.374 (7)
N1—C17	1.479 (6)	C19—H19A	0.9300
C1—C3^ii^	1.386 (6)	C20—C21	1.368 (8)
C1—C2	1.387 (6)	C20—H20A	0.9300
C1—H1A	0.9300	C21—C22	1.364 (7)
C2—C3	1.396 (6)	C21—H21A	0.9300
C3—C1^ii^	1.386 (6)	C22—C23	1.378 (7)
C3—H3A	0.9300	C22—H22A	0.9300
C4—H4A	0.9700	C23—H23A	0.9300
C4—H4B	0.9700	C24—C25	1.378 (7)
C5—C10	1.400 (7)	C24—C29	1.386 (7)
C5—C6	1.405 (7)	C25—C26	1.404 (7)
C6—C7	1.352 (8)	C25—H25A	0.9300
C6—H6A	0.9300	C26—C27	1.342 (8)
C7—C8	1.377 (9)	C26—H26A	0.9300
C7—H7A	0.9300	C27—C28	1.358 (8)
C8—C9	1.387 (9)	C27—H27A	0.9300
C8—H8A	0.9300	C28—C29	1.380 (7)
C9—C10	1.368 (8)	C28—H28A	0.9300
C9—H9A	0.9300	C29—H29A	0.9300
			
P1—Cu1—Cl2	127.78 (6)	C16—C11—C12	117.7 (5)
P1—Cu1—Cl1	125.05 (7)	C16—C11—P1	117.4 (4)
Cl2—Cu1—Cl1	93.71 (7)	C12—C11—P1	124.5 (4)
P1—Cu1—Cl1^i^	109.03 (6)	C13—C12—C11	122.2 (6)
Cl2—Cu1—Cl1^i^	102.38 (6)	C13—C12—H12A	118.9
Cl1—Cu1—Cl1^i^	91.68 (6)	C11—C12—H12A	118.9
P2^ii^—Cu2—Cl2	134.50 (6)	C12—C13—C14	118.9 (7)
P2^ii^—Cu2—Cl1	126.84 (6)	C12—C13—H13A	120.5
Cl2—Cu2—Cl1	98.57 (7)	C14—C13—H13A	120.5
Cu2—Cl1—Cu1	81.73 (6)	C15—C14—C13	120.7 (6)
Cu2—Cl1—Cu1^i^	115.71 (6)	C15—C14—H14A	119.6
Cu1—Cl1—Cu1^i^	88.32 (6)	C13—C14—H14A	119.6
Cu2—Cl2—Cu1	82.49 (7)	C14—C15—C16	119.5 (6)
C5—P1—C11	109.2 (2)	C14—C15—H15A	120.2
C5—P1—C4	97.5 (2)	C16—C15—H15A	120.2
C11—P1—C4	100.9 (2)	C11—C16—C15	120.8 (6)
C5—P1—Cu1	116.30 (18)	C11—C16—H16A	119.6
C11—P1—Cu1	113.65 (18)	C15—C16—H16A	119.6
C4—P1—Cu1	117.26 (16)	N1—C17—P2	111.4 (3)
C18—P2—C24	106.1 (2)	N1—C17—H17A	109.4
C18—P2—C17	102.1 (2)	P2—C17—H17A	109.4
C24—P2—C17	98.1 (2)	N1—C17—H17B	109.4
C18—P2—Cu2^ii^	117.10 (16)	P2—C17—H17B	109.4
C24—P2—Cu2^ii^	118.43 (17)	H17A—C17—H17B	108.0
C17—P2—Cu2^ii^	112.24 (17)	C23—C18—C19	117.1 (4)
C2—N1—C4	121.7 (4)	C23—C18—P2	118.3 (4)
C2—N1—C17	119.9 (4)	C19—C18—P2	124.6 (4)
C4—N1—C17	118.2 (4)	C20—C19—C18	121.5 (5)
C3^ii^—C1—C2	121.7 (4)	C20—C19—H19A	119.2
C3^ii^—C1—H1A	119.1	C18—C19—H19A	119.2
C2—C1—H1A	119.1	C21—C20—C19	120.2 (5)
C1—C2—C3	117.0 (4)	C21—C20—H20A	119.9
C1—C2—N1	121.9 (4)	C19—C20—H20A	119.9
C3—C2—N1	121.1 (4)	C22—C21—C20	119.3 (5)
C1^ii^—C3—C2	121.2 (4)	C22—C21—H21A	120.3
C1^ii^—C3—H3A	119.4	C20—C21—H21A	120.3
C2—C3—H3A	119.4	C21—C22—C23	120.6 (5)
N1—C4—P1	112.3 (3)	C21—C22—H22A	119.7
N1—C4—H4A	109.1	C23—C22—H22A	119.7
P1—C4—H4A	109.1	C22—C23—C18	121.2 (5)
N1—C4—H4B	109.1	C22—C23—H23A	119.4
P1—C4—H4B	109.1	C18—C23—H23A	119.4
H4A—C4—H4B	107.9	C25—C24—C29	118.2 (5)
C10—C5—C6	116.0 (5)	C25—C24—P2	117.9 (4)
C10—C5—P1	117.8 (4)	C29—C24—P2	123.6 (4)
C6—C5—P1	125.0 (4)	C24—C25—C26	119.8 (5)
C7—C6—C5	123.2 (6)	C24—C25—H25A	120.1
C7—C6—H6A	118.4	C26—C25—H25A	120.1
C5—C6—H6A	118.4	C27—C26—C25	120.8 (5)
C6—C7—C8	118.8 (6)	C27—C26—H26A	119.6
C6—C7—H7A	120.6	C25—C26—H26A	119.6
C8—C7—H7A	120.6	C26—C27—C28	120.1 (6)
C7—C8—C9	120.8 (6)	C26—C27—H27A	120.0
C7—C8—H8A	119.6	C28—C27—H27A	120.0
C9—C8—H8A	119.6	C27—C28—C29	120.5 (6)
C10—C9—C8	119.4 (6)	C27—C28—H28A	119.8
C10—C9—H9A	120.3	C29—C28—H28A	119.8
C8—C9—H9A	120.3	C28—C29—C24	120.6 (5)
C9—C10—C5	121.7 (6)	C28—C29—H29A	119.7
C9—C10—H10A	119.1	C24—C29—H29A	119.7
C5—C10—H10A	119.1		

Symmetry codes: (i) −*x*−1, −*y*, −*z*+1; (ii) −*x*, −*y*, −*z*+1.
